# Molecular anatomy of the receptor binding module of a bacteriophage long tail fiber

**DOI:** 10.1371/journal.ppat.1008193

**Published:** 2019-12-19

**Authors:** Mohammad Z. Islam, Andrei Fokine, Marthandan Mahalingam, Zhihong Zhang, Carmela Garcia-Doval, Mark J. van Raaij, Michael G. Rossmann, Venigalla B. Rao

**Affiliations:** 1 Department of Biology, The Catholic University of America, Washington, DC, United States of America; 2 Department of Biological Sciences, Purdue University, West Lafayette, Indiana, United States of America; 3 Centro Nacional de Biotecnologia, Consejo Superior de Investigaciones Cientificas, Madrid, Spain; The Scripps Research Institute, UNITED STATES

## Abstract

Tailed bacteriophages (phages) are one of the most abundant life forms on Earth. They encode highly efficient molecular machines to infect bacteria, but the initial interactions between a phage and a bacterium that then lead to irreversible virus attachment and infection are poorly understood. This information is critically needed to engineer machines with novel host specificities in order to combat antibiotic resistance, a major threat to global health today. The tailed phage T4 encodes a specialized device for this purpose, the long tail fiber (LTF), which allows the virus to move on the bacterial surface and find a suitable site for infection. Consequently, the infection efficiency of phage T4 is one of the highest, reaching the theoretical value of 1. Although the atomic structure of the tip of the LTF has been determined, its functional architecture and how interactions with two structurally very different *Escherichia coli* receptor molecules, lipopolysaccharide (LPS) and outer membrane protein C (OmpC), contribute to virus movement remained unknown. Here, by developing direct receptor binding assays, extensive mutational and biochemical analyses, and structural modeling, we discovered that the ball-shaped tip of the LTF, a trimer of gene product 37, consists of three sets of symmetrically alternating binding sites for LPS and/or OmpC. Our studies implicate reversible and dynamic interactions between these sites and the receptors. We speculate that the LTF might function as a “molecular pivot” allowing the virus to “walk” on the bacterium by adjusting the angle or position of interaction of the six LTFs attached to the six-fold symmetric baseplate.

## Author summary

Bacteriophage (phage) T4 belongs to myoviridae, a widely distributed family of viruses on Earth. They contain a head (capsid), a contractile tail, and a baseplate to which six long tail fibers (LTFs) are attached. During infection, the genome packed inside the capsid is injected into its host, *Escherichia coli* bacterium, to initiate virus replication. The first step of infection is recognition of receptor molecules, lipopolysaccharide (LPS) and/or outer membrane protein C (OmpC), present on bacterial surface by the tips of LTFs. This allows phage to attach to bacterium, move on the surface, and find a suitable site for infection. However, the interactions that govern this critical process are poorly understood. Here, we provide the first molecular description of a tail fiber tip. Extensive mutational, structural, and biochemical analyses show that the ball-shaped tip contains patches of binding sites that allow dynamic interactions with LPS and/or OmpC. We speculate that each LTF might act as a molecular pivot, able to change its position and angle and allow phage to move on the bacterium. Our studies uncover the basic architecture of a phage molecular device used for gaining entry into bacteria and provide insights into engineering novel phages to curtail multidrug-resistance bacteria.

## Introduction

Bacteriophages (phages) are the most numerous biological entities on Earth [1, 2]. Yet, the molecular details of initial interactions between a phage and a bacterium that then lead to irreversible virus attachment and genome ejection remained unknown. Elucidation of these interactions is critical not only to understand how the phage infection machines operate but also to engineer these machines to kill multi-drug resistant bacteria [3]. There is particular urgency in this area due to the emergence of rampant antibiotic resistance, one of the biggest threats to global health today[4].

**Fig 1:**
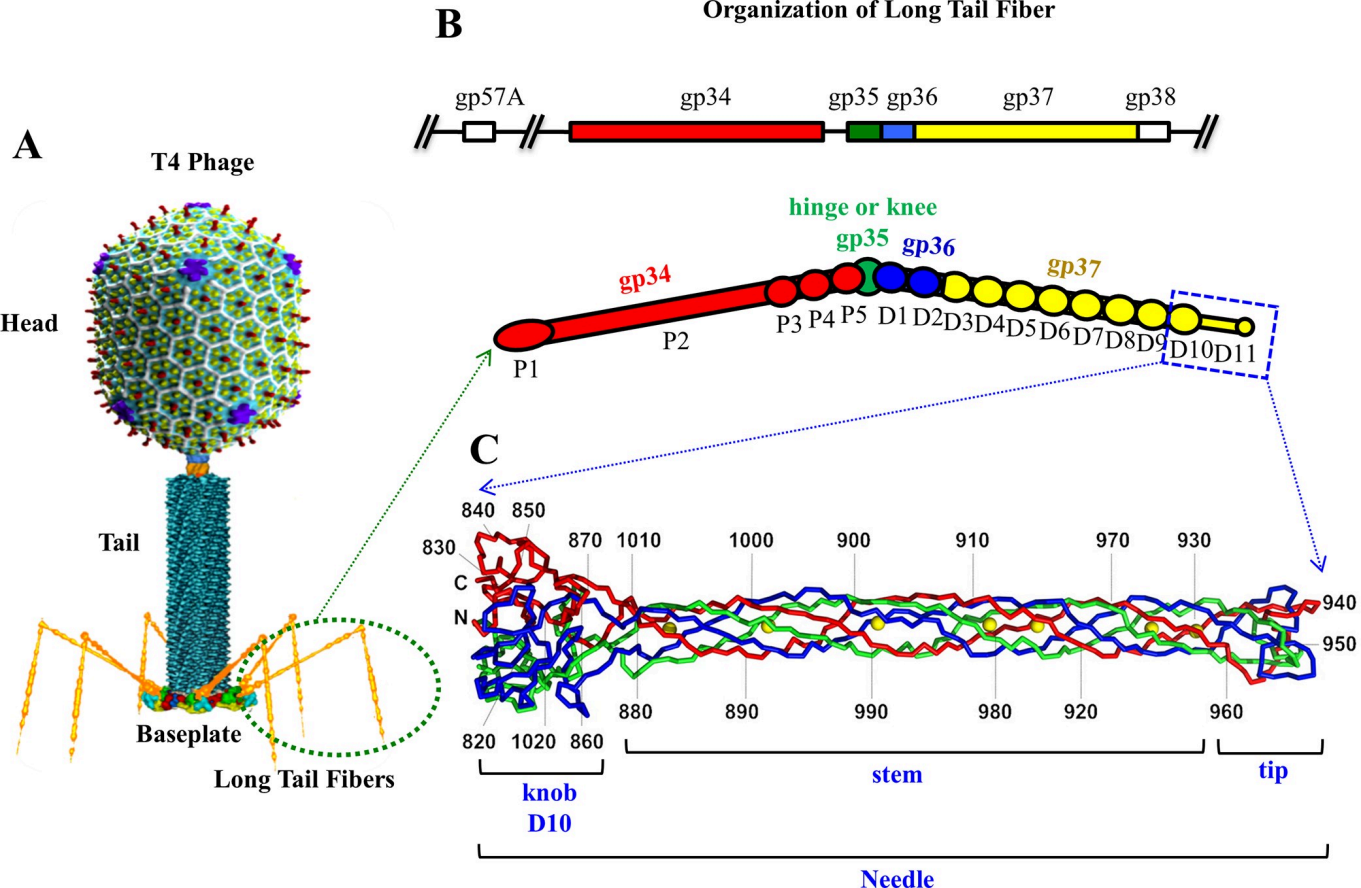
Organization of the bacteriophage T4 long tail fiber. (A) A structural model of bacteriophage T4 virion showing the head, the tail, and the long tail fibers. (B) The proximal half of the LTF is formed by gp34 trimer (red), the knee cap is formed by gp35 monomer (green), and the distal half is formed by gp36 trimer (blue) and gp37 trimer (yellow) [10]. The part of the gp37 trimer for which the X-ray structure was determined is outlined by blue rectangle. (C) The crystal structure of the gp37 C-terminal fragment [18]. The three polypeptide chains in the gp37 trimer are shown in red, blue, and green. The ferrous ions are shown as yellow spheres.

Bacteriophage T4 is a well-studied tailed, double-stranded, DNA virus that belongs to the family *Myoviridae* of the order *Caudovirales*, which includes ~95% of all phages visualized by electron microscopy (EM) [5] (Fig 1). With ~168 kbp dsDNA genome containing 289 open reading frames, T4 encodes 40 structural proteins that form the infectious virion [6]. The T4 virion has a 1150 Å-long and 850 Å-wide prolate head encapsidating the genome, and a 1200 Å-long contractile tail (Fig 1) [7]. The tail contains an internal rigid tube, surrounded by a contractile sheath, and a baseplate located at the distal end [8–11]. The T4 virion also contain two types of fibers: six short tail fibers (STFs), which are folded beneath the baseplate, and six long tail fibers (LTFs), attached to the baseplate’s periphery and extended outward. The STFs and LTFs have lengths of around 400 Å and 1450 Å, respectively [12] (Fig 1A).

With two sets of tail fibers, T4 phage possesses one of the most effective infection machines known with an infection efficiency close to the theoretical value of 1 [13]. LTFs play a critical role, being responsible for the initial host cell recognition and initiation of infection. Each LTF contains two long, thin, and rigid rods (half-fibers), attached to each other via a hinge or knee joint (Fig 1B). The LTF is assembled from ten polypeptide chains of four different gene products (gps); gp34 (140 kDa), gp35 (35 kDa), gp36 (23 kDa) and gp37 (109 kDa) [14]. The rod proximal to the baseplate is formed by a homotrimer of gp34 [15]. The hinge is formed by monomeric gp35, whereas the distal rod is formed by homotrimers of gp36 and gp37 [16]. Based on EM studies [16], the density of the distal half of the fiber was subdivided into ten globular “knob” domains, D1–D10, and an elongated domain, D11, at the distal end of the LTF. Domains D1 and D2 located near the hinge are probably made of gp36, whereas domains D3–D11 are formed by gp37 (Fig 1C). LTF assembly is assisted by the chaperone protein gp57A, which helps in the trimerization of gp34 and gp37, whereas the chaperone protein gp38 is required for proper folding of gp37 [17].

The structure of the gp37 C-terminal region (“needle”) containing domains D10 and D11 has been determined by X-ray crystallography (Fig 1C) [18]. The D10 domain (residues 811–881 and 1010–1026) has the same fold as the “collar” domain of the short tail fiber protein, gp12, and also regions of the baseplate proteins gp10 and gp11, suggesting that these proteins may have a common evolutionary origin. The elongated domain D11, formed by residues 882–1009, is an insertion into domain D10. The crystal structure suggests that the D11 domain can be further subdivided into a “stem” subdomain (residues 882–931 and 960–1009) and a smaller “tip” subdomain (residues 932–959) (Fig 1C). The tip subdomain, which itself is an insertion into the stem subdomain, forms the distal pole of the fiber, which probably contains binding sites for the host receptors, lipopolysaccharide (LPS) and outer membrane protein C (OmpC).

Early genetic studies implicated the *Escherichia coli* cell surface molecules LPS and OmpC as the host receptors for phage T4. T4 can efficiently infect *E*. *coli* strains B and K12. However, strain B lacks OmpC, therefore, only LPS molecules can be used as receptors. The LPS of strain B have two terminal glucose (Glu) residues, Glu I and Glu II (S1 Fig) [19–21]. Previous studies [20–23] suggest that the T4 LTF might interact with Glu I or with both Glu I and Glu II. The K12 strain contains both LPS and OmpC, however its Glu I and Glu II of LPS are modified by linkage to additional sugar residues, which probably interfere with the binding of LTF to Glu I and Glu II. Hence, interaction with OmpC is also needed for efficient adsorption of T4 to K12 *E*. *coli* [24, 25].

Phage T4, and other tailed phages such as T7, λ, and Sf6, are thought to “walk” on the bacterium using the LTFs as “legs” [26–31]. Walking allows the virus to scan the large surface area of a bacterial cell and find a suitable site for infection. This might also be a general phenomenon in many other phages and viruses but is poorly understood. In the case of phage T4, the “poles” of the rod-shaped *E*. *coli* bacterium are reported to be enriched with the infection sites [28]. LTFs not only provide a means to reach these sites but also signal the baseplate inducing a series of conformational changes. The short tail fibers unravel and irreversibly attach to their receptors which then leads to tail sheath contraction, penetration of tail tube, and genome ejection [8, 9, 11, 32–36]. A fundamental question that arises, and remains unresolved, is: what is the functional architecture of the LTF that allows virus movement on bacterial surface? In fact, despite solving numerous atomic structures of tail fibers from different phages [37, 38]), the interactions between a phage tail fiber and its receptor(s) are poorly understood. This knowledge is critical to engineer novel host specificities to combat antibiotic resistance.

Here, by developing new and direct receptor binding assays, extensive genetic and biochemical analyses, and structural modeling, we have delineated the molecular anatomy of the receptor-binding module of the LTF of phage T4. Our results show that the tip of the LTF consists of patches of LPS and/or OmpC binding specificity symmetrically displayed around the ball of the tip. This architecture creates a “molecular pivot” that is able to turn in its receptor “cavity” through reversible interactions, allowing the LTFs to adjust their angles and/or positions and move on the bacterial surface. These studies provide the first detailed description of a specialized molecular device evolved to maximize the infection efficiency of a tailed phage.

## Results

### Phage T4 LTF needle binds to both LPS and OmpC receptors

Although LPS and OmpC were known to be the host receptors for phage T4 [39–42], it is unknown if LTF alone can interact with both these receptors. If it does, it is unclear how it recognizes two structurally very different molecules. LPS is a fibrous lipopolysaccharide whereas OmpC is a transmembrane protein pore [43]. To resolve this question and dissect the molecular interactions, we have developed direct binding assays using purified components.

**Fig 2:**
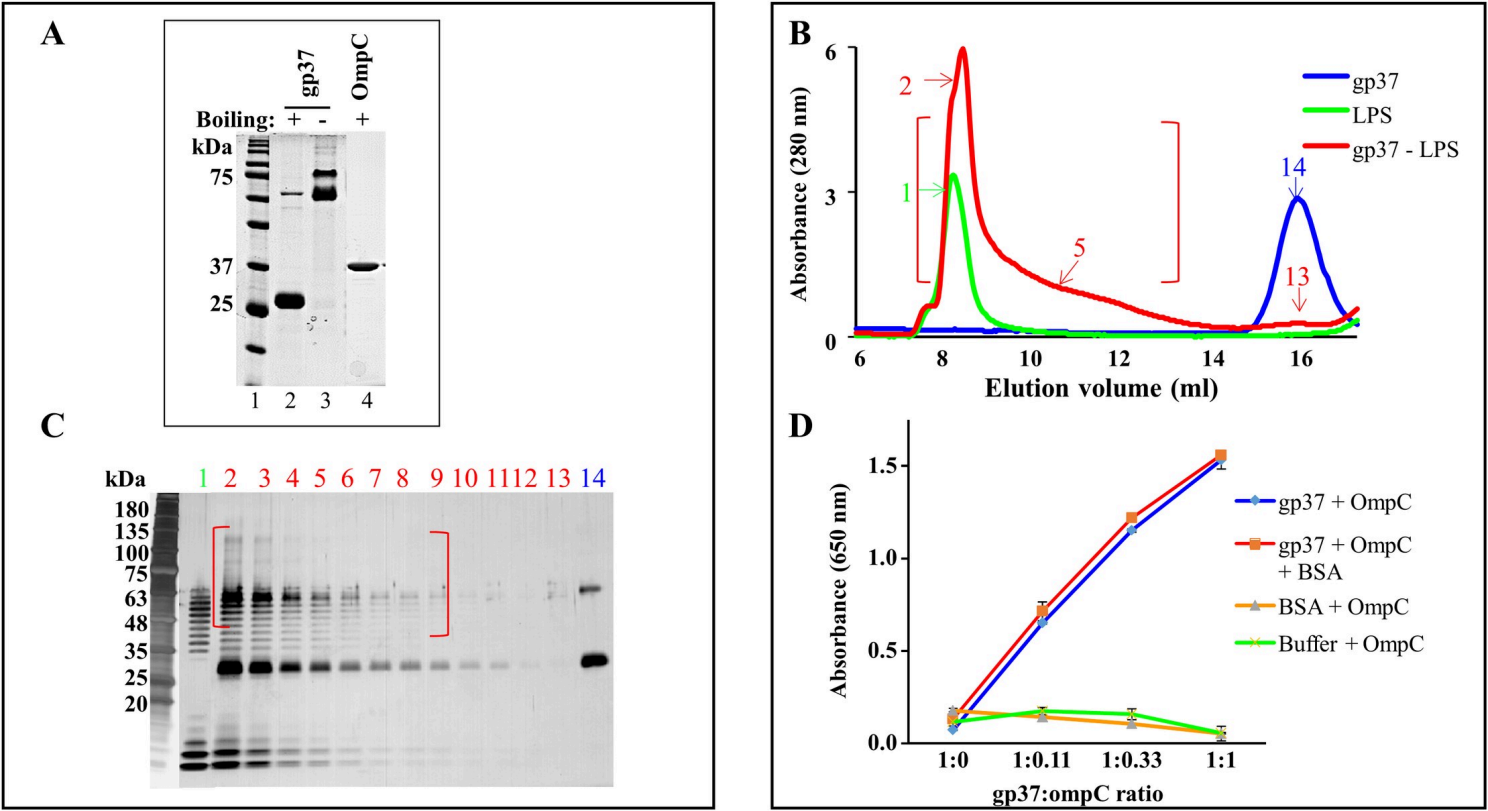
The LTF needle binds to both LPS and OmpC receptors. (A) SDS-PAGE profiles of purified LTF needle and OmpC trimers. “+” and “-” represent boiling in the presence of SDS. The boiled gp37 sample (lane 2) showed the major 27 kDa monomer band and a faint band of residual trimer present in the boiled sample. The unboiled gp37 sample (lane 3) showed two trimer bands, which probably represent two trimer conformations, intact trimer and partially unfolded trimer. (B) Diazirine-labelled gp37 was incubated with purified LPS from *E*. *coli* B for 1 h at 37ºC followed by UV irradiation for 15 min and Superose 6 10/300 GL size-exclusion chromatography (red). Same amounts of diazirine-labelled gp37 (blue) and LPS (green) were loaded on the same column as controls. (C) SDS-PAGE profile of the peak fractions from panel B. The gel was developed by silver staining. Lanes 2 to 13 (elution volumes corresponding to 8 to 16 ml) show fractions of gp37-LPS complex peak. Lane 1 shows LPS control peak (elution volume, 9 ml) and lane 14 shows the gp37 control peak (elution volume, 16 ml). Note the presence of a small amount of cross-linked dimer of gp37 of trimers in lane 14. (D) ELISA assay to capture gp37-OmpC complex. The wells were coated with gp37 and OmpC was added at various molar ratios. The unbound OmpC was removed by repeated washing and the amount of bound OmpC was determined by adding mouse anti-OmpC antibody followed by HRP-conjugated anti- mouse IgG antibody. Various controls (coating buffer + OmpC; BSA + OmpC; or gp37 + OmpC + BSA) were included to validate the specific interaction between gp37 and OmpC.

We first purified the LTF needle (gp37; amino acids 799–1026) containing the distal-most tip subdomain which likely interacts with the receptors. Recombinant clones were constructed with a hexa-histidine tag attached to the N-terminus of the gp37 needle sequence. The protein was over-expressed in *E*. *coli* along with co-expression of its chaperones gp38 and gp57A. The needles were then purified by Ni-affinity and size-exclusion chromatographies [44]. The gp37 needle protein formed trimers and oligomers. The trimers, which eluted at the expected 81 kDa size were pooled and tested for binding to LPS and OmpC. These trimers were stable in SDS at room temperature but dissociated into monomers at boiling temperature (Fig 2A), a characteristic feature of the LTF trimer [44] that indicated that the heterologously expressed LTF needles assembled into native-like structures.

Several assay formats were tested to determine the binding of gp37 needle to purified LPS from *E*. *coli* B cells. The results indicated that they do form a complex but it is unstable, consistent with a previous report where an LPS extract was used [24]. We therefore optimized a mild crosslinking protocol to stabilize the needle-LPS complex. The LTF needle was pre-treated with the crosslinking reagent diazirine (NHS-LC-Diazirine) such that the reagent covalently attaches to the trimer but does not cause significant inter-trimer crosslinking, as determined by gel electrophoresis. The diazirine-modified gp37 was then incubated with LPS and exposed to UV light to crosslink and stabilize the needle-LPS complex, which was then separated by Superose-6 size-exclusion chromatography (Fig 2B). As controls, the same amounts of unlinked LPS or diazirine-gp37 were independently passed through the same column. Comparison of the elution profiles demonstrated that the gp37 needle peak disappeared completely upon incubation with LPS and a broad peak appeared near the void volume. Since LPS is an elongated fiber whereas the LPS-gp37 complex will have a more complex shape, the latter showed a broad and anomalous elution behavior on the size-exclusion column (Fig 2B, shown in red). This new peak contained crosslinked bands in addition to the gp37 band and a ladder of LPS bands (the ladder is due to different numbers of repeating sugar units linked to LPS [45]. On the other hand, in the controls, only the LPS ladder (lane 1) or the gp37 (lane 14) bands were seen in the respective peak fractions (Fig 2C). Furthermore, no gp37 peak was seen at the position of the LPS-gp37 complex in the gp37 control (Fig 2B, shown in blue). These data demonstrated that the shift of gp37 and broadening of the LPS peak near the void volume of size-exclusion column were due to the formation of gp37-LPS complexes.

For testing the binding of gp37 needles to OmpC, recombinant OmpC protein was over-expressed in *E*. *coli* BZB1109 and purified by selective extraction of membrane-associated OmpC with octyl-polyoxy ethylene detergent and separation of soluble OmpC by chromatofocusing and size-exclusion chromatography [43]. We then raised OmpC-specific polyclonal antibodies in mice using the purified OmpC protein. Quantification by ELISA showed that the polyclonal serum contained very high titers of OmpC-specific antibodies (>10^7^ endpoint titer) (S2 Fig). A direct binding assay was then developed in which the LTF needles were coated on a 96-well plate and allowed to bind to OmpC trimers. After washing off the unbound OmpC, the bound OmpC was quantified using the OmpC polyclonal antibodies and a peroxidase-coupled second antibody. Considering that gp37 binds weakly to LPS and that OmpC is not an essential receptor for T4, weaker binding was expected. However, surprisingly, the LTF needle bound strongly to OmpC trimer in a concentration dependent manner and no significant nonspecific binding was evident (Fig 2D).

### Amino acids lining the LTF needle tip interact with LPS and OmpC receptors

**Fig 3:**
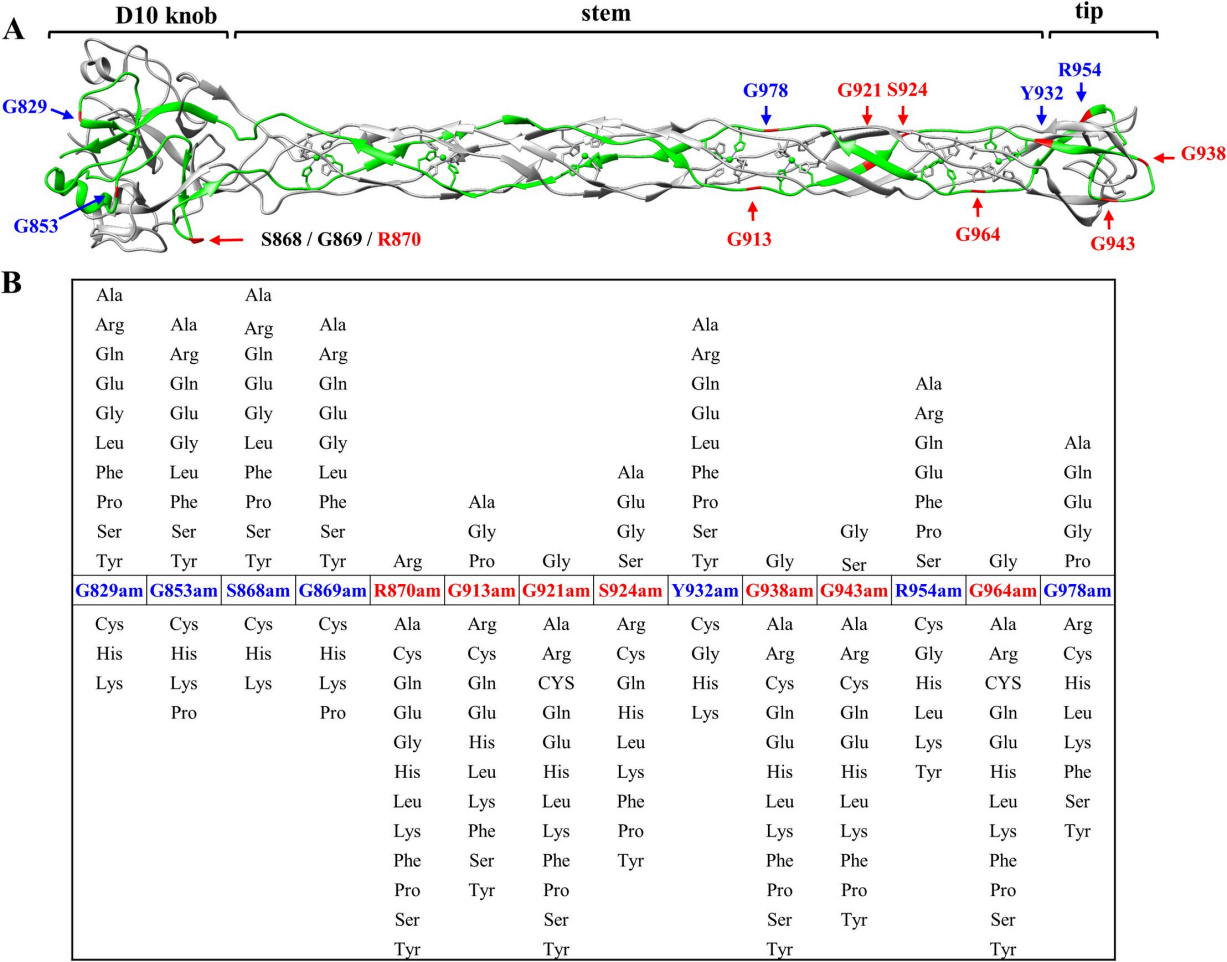
Mutational analysis of the LTF needle. (A) gp37 trimer with one of the polypetide chains highlighted in green. The positions of amino acids on the backbone where an amber stop codon was introduced are shown in red and blue. (B) Suppressor patterns of fourteen gp37 amber mutant phages, each tested with 13 different suppressor *E*. *coli* strains. Amino acid substitutions above the native sequence represent functional phenotypes and those below represent lethal phenotypes. Red numbers and letters in both A and B represent the amino acids that are essential for function and blue numbers and letters represent the amino acids that are not essential for function.

To determine which amino acids of the LTF needle interact with the receptors, a series of fourteen amber stop codon mutations were introduced into D10 knob, stem, and tip subdomains of the LTF needle (Fig 3A). Each amber mutant phage was then tested on 13 different amber suppressor *E*. *coli* strains, each incorporating a different amino acid at the amber termination codon [46]. If the substituted amino acid is tolerated, the mutant phage will produce plaques, otherwise no plaques will appear. The amber mutants, in addition, provided genetic markers along the length of the needle for high-resolution mapping (see below). The data showed that several glycines in the stem and tip subdomains are essential for function (Fig 3B). The stem glycines G921 and G964 are most likely required for protein folding and/or structural integrity of the LTF trimer. G921 is located in a β-strand and interacts with F970 from another gp37 subunit (Fig 3A). The side chain of F970 forms the hydrophobic core of the gp37 trimer. Structural analyses predict that substitution of G921 with any other residue would clash with the F970 sidechain and may interfere with the formation of the hydrophobic core. G964 is in a loop region that interacts with H929 of a neighboring gp37 subunit. The three H929 residues of the trimer form the metal binding motif that coordinates with an iron atom that is critical for LTF folding and structure. There are seven such coordinated iron atoms along the length of the needle domain (Fig 1C). The structure [18] predicts that any other sidechain at the G964 position would clash with H929, likely disturbing the iron binding site.

**Fig 4:**
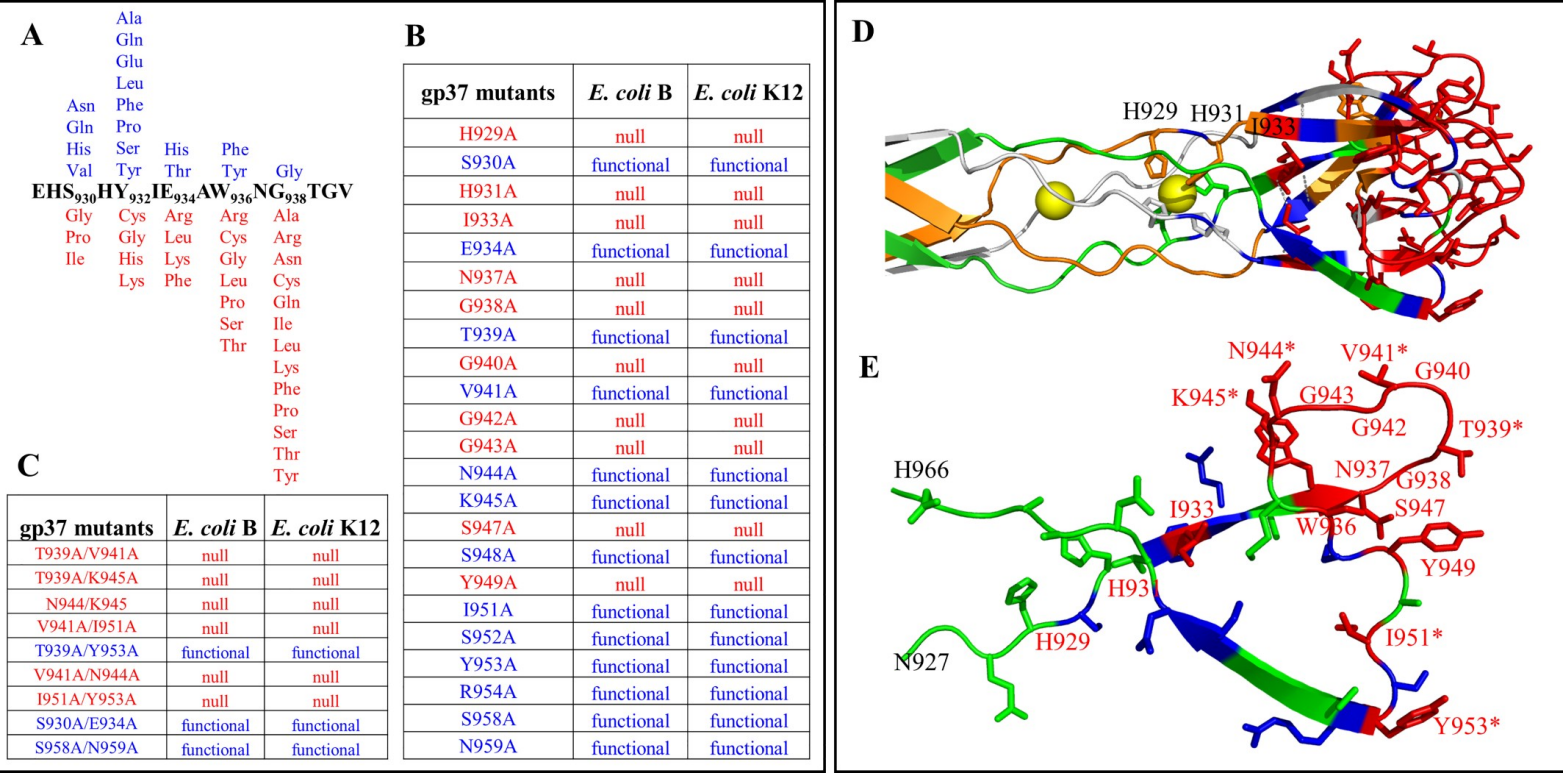
Amino acids lining the LTF tip surface interact with LPS and OmpC receptors. (A) Combinatorial mutagenesis was used to determine the functional importance of each of the amino acids. See S3 Fig for a schematic of combinatorial mutagenesis and genetic rescue strategies. Amino acid substitutions shown above the native sequence represent functional phenotype (blue) and the ones below (red) represent null phenotype. (B & C) Functional importance of the alanine substitutions. Residues occupying the tip of the LTF were tested for their functional importance by constructing single (B) and double (C) alanine substitution mutants followed by genetic rescue. Phenotypes were scored as functional (blue) or null (red). (D) Critical single residues identified in marker rescue assays are highlighted in red. “*” represents the amino acids that were found to be null in the background of double alanine substitutions. (E) A single polypeptide chain showing functionally important (red) or nonessential (blue) amino acid residues.

The tip subdomain residues G938 and G943 are in a loop that lines the distal surface of the tip. Both these glycines are critical for function since no amino acid substitutions except for a serine at G943 were tolerated (Fig 3B). Mutation of these glycines, therefore, probably interfered with the receptor binding function of the tip. To analyze this further, we have performed high-resolution functional mapping by constructing combinatorial libraries (S3 Fig) at amino acids S930, Y932, E934, W936, and G938 that are linked to the same loop (Fig 4A). All possible codons were introduced at each of these amino acids and each mutation was transferred into T4 genome by recombinational rescue using the amber mutant phages constructed above. Of several hundred random mutants tested from each library, 54.5% of S930 mutants, 75% of Y932, 30% of E934 mutants, 12% of W936 mutants, and 7% of G938 mutants were functional phenotypes producing plaques. Sequencing of dozens of these mutants demonstrated that residues W936 and G938 are critical for function (Fig 4A). No substitutions including an alanine were tolerated at G938 whereas only aromatic substitutions such as Phe or Tyr were tolerated at W936 residue.

We then extended the functional mapping to virtually every amino acid of the tip subdomain by substituting each amino acid with alanine and testing its phenotype by the above genetic rescue strategy (Fig 4B). The data showed that the iron-binding histidines, as well as many of the residues lining the surface of the tip are critical for function. These include H929, H931, I933, N937, G938, G940, G942, G943, S947, and Y949. Alanine substitution at any of these residues resulted in complete loss of plaque forming ability. On the other hand, alanine substitution at residues T939, V941, N944, K945, S948, I951, S952, Y953, R954, S958, and N959 retained plaque forming ability. To determine if this tolerance was because single amino acid substitution was insufficient to completely disrupt function, we constructed a series of double alanine substitutions at these residues and tested their plaque forming ability. We found that many of these amino acids indeed turned out to be important when two amino acids were simultaneously mutated (Fig 4C). These include double mutants T939A-V941A, T939A-K945A, V941A-I951A, I951A-Y953A, and N944A-K945A, which produced no plaques.

Together, the above mutational data suggest that essentially the entire surface of the LTF tip is important for function (Fig 4D & 4E). Furthermore, insertion of even a single amino acid at any point on this surface resulted in lethality. Remarkably, most of the mutants exhibited all-or-none behavior when they were tested on *E*. *coli* B and K12 strains. Each mutant that lost plaque-forming ability on *E*. *coli* B strain containing the wild-type (WT) LPS receptor but lacking OmpC also lost the plaque-forming ability on *E*. *coli* K12 strain which has modified LPS but containing the OmpC receptor (Figs 4B, 4C and S1). This indicated that the same binding site might be involved in interacting with both the receptors, LPS and OmpC, although these are two structurally very different molecules.

### Modulation of LTF interactions with LPS and OmpC

**Fig 5:**
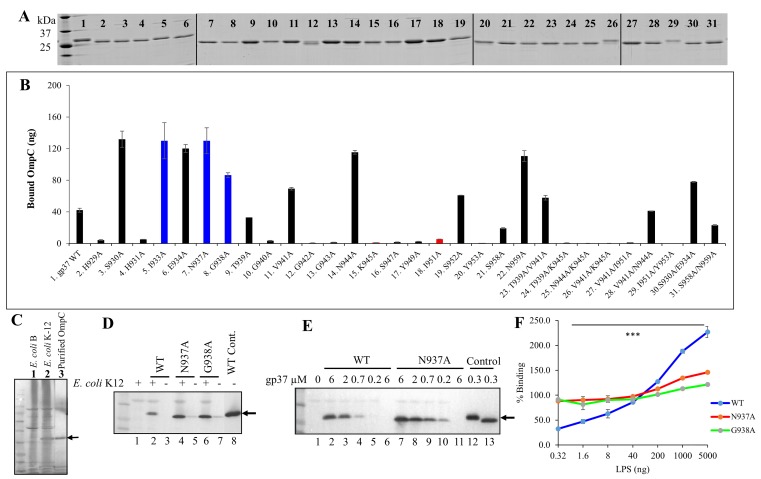
Modulation of LTF-receptor interactions. (A) SDS-PAGE of purified mutant LTF needle proteins. The first lane on the left show molecular mass standards. A vertical line was added between images that were sliced and pasted. (B) Quantification of LTF needle-OmpC interaction by direct binding ELISA assay. Binding results that correlated with the *in vivo* genetic assays (Fig 4) are shown as black bars and those that did not correlate are shown either as blue bars (greater binding than WT needle) or red bars (poorer binding than WT needle). (C) Western blot with anti-OmpC antibody showing the presence of OmpC band in the *E*. *coli* K12 strain (lane 2), but not in the B strain (lane 1). The purified OmpC protein was included in lane 3 as a positive control. (D) Binding of WT and mutant LTF needle proteins to *E*. *coli*. Bound gp37 was detected by Western blotting using monoclonal anti-histag antibodies. Lane 1 is negative control in which the gp37 protein was omitted. Lanes 3, 5, and 7 represent negative controls where *E*. *coli* was omitted. Lane 8 is the positive control where purified WT gp37 was loaded into the well. (E) Dependence of protein concentration on the binding of WT and N937A gp37 proteins to *E*. *coli* K12 bacteria. Lane 1 is the negative control where the gp37 protein was omitted and lanes 6 and 11 are negative controls where *E*. *coli* was omitted. Arrows show the position of the gp37 band. (F) Binding of WT gp37, N937A, and G938A proteins to *E*. *coli* bacteria in the presence of increasing concentrations of purified *E*. *coli* B LPS.

To analyze the LPS and OmpC interactions directly, we constructed expression clones for most of the above mutations and purified more than thirty different mutant LTF needles (Fig 5A). All the mutant proteins could form native-like trimers. Presumably, since the mutations are in the loop regions, they did not abrogate gp37 folding. The mutant trimers were then tested for their ability to bind OmpC using the direct ELISA binding assay described above (Fig 2D). Consistent with the genetic data, most of the mutants that lost plaque forming ability on WT LPS^+^ *E*. *coli* B strain also lost the ability to bind OmpC (Fig 5B).

However, there are exceptions. Three of the mutant needles, I933A, N937A, and G938A bound 2–3 fold more efficiently to OmpC than the WT needle, yet these mutants completely lost plaque forming ability on either OmpC^-^ or OmpC^+^ *E*. *coli* strains. To analyze if this is the case in a native context, we have tested the binding of mutant needles to whole *E*. *coli* cells (Fig 5C & 5D). The mutant proteins were incubated with OmpC^-^ or OmpC^+^ *E*. *coli* and the cell-bound gp37 was quantified by Western blotting after washing off the unbound gp37. These data showed that, consistent with the direct binding data, the N937A and G938A mutant needles bound to OmpC^+^ *E*. *coli* 2–3 fold more efficiently than the WT needle (Fig 5D & 5E). We then tested if the presence of LPS affected OmpC binding. The data showed that LPS enhanced the binding of WT LTF needle to OmpC in a concentration dependent manner whereas the mutant needle was insensitive to LPS. It bound at near maximum level in the absence of LPS and showed only a slight enhancement at very high concentrations of LPS (Fig 5F). These data suggested that the binding of WT needle to OmpC, but not of the mutant needle, was modulated by LPS. Thus, the mutants appeared to have lost LPS interaction but gained in OmpC binding, probably making the LTF less dynamic in its interactions with receptors, costing in phage’s ability to form plaques (Fig 4B).

Another exception was that certain mutants showed a gain-of-function phenotype. Mutants K945A, I951A and Y953A lost the ability to bind OmpC (Fig 5B) but retained LPS binding as evident by their ability to form plaques on *E*. *coli* B strain (Fig 4B). Surprisingly, however, these mutants formed plaques on *E*. *coli* K12 strain which normally requires OmpC binding. This means that these mutants, unlike the WT LTF, can use the K12 LPS receptor more effectively to compensate for loss of OmpC binding, even though the Glu I and Glu II of K12 LPS are masked by linkage with other sugars (S1 Fig).

## Discussion

### Architecture of the LTF tip

**Fig 6:**
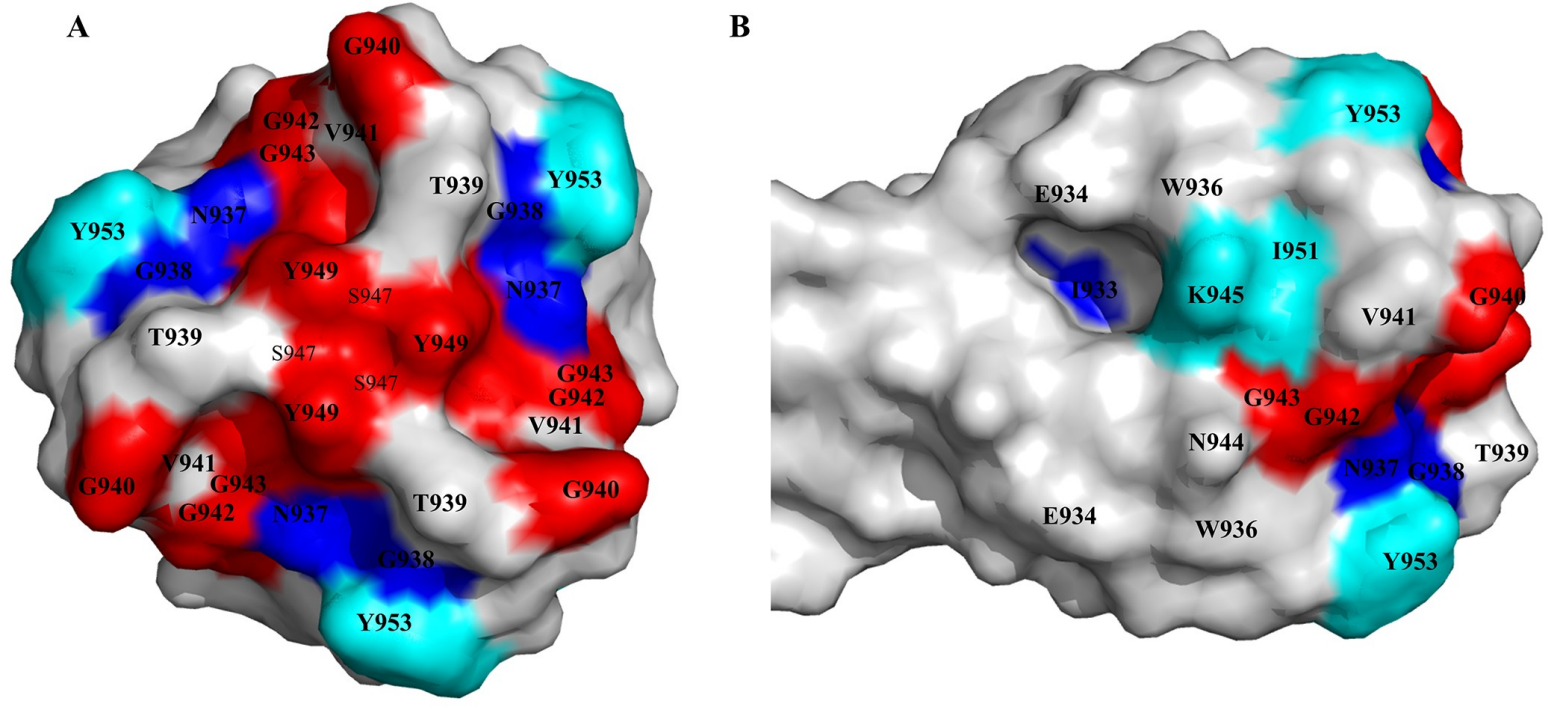
LPS and OmpC receptor binding patches on the LTF tip. Surface representation of the LTF tip showing top (A) and side (B) views with the functionally critical amino acid residues highlighted. Red patch corresponds to amino acids that are important for both LPS and OmpC binding, blue patch for LPS binding, and cyan patch for OmpC binding.

Our mutational and biochemical studies show remarkable clustering of host receptor interacting amino acid residues at the tip of the phage T4 long tail fiber. Based on phenotypic behavior, they correspond to three patches of receptor specificity (Fig 6; S1 Movie); one for interaction with both LPS and OmpC receptors (red), another for binding the LPS receptor (blue), and a third one for binding the OmpC receptor (cyan). Since there are three molecules of gp37 in each fiber, there are nine symmetrically arranged patches of specificity encircling the “ball” of the trimeric tip.

Early studies suggested that LTF binds to the terminal glucose residues of *E*. *coli* B LPS [20–22]. The crystal structure of the needle showed that there are three small cavities at the bottom of the tip [18]. Residues in the first patch of specificity; G940, G942, G943, S947 and Y949, occupy part of this cavity, the bottom-most portion facing the bacterial surface when the T4 phage lands on the *E*. *coli* bacterium (S2 Movie). These residues are critical for LPS interaction, and the volume of this cavity is suitable for accommodation of one glucose moiety. The aromatic ring of the Y949 residue projects out at the bottom rim of the cavity and is in good position to form stacking interactions with the terminal glucose of LPS. Docking of LPS glucose onto the gp37 tip [47] produced docking solutions with the glucose placed in this cavity. Therefore, this cavity is likely to be the binding site for the terminal LPS glucose residue(s). Since the gp37 trimer has three such cavities, LTF can potentially bind up to three LPS molecules.

The above amino acids were also found to be important for OmpC binding. Docking of the gp37 needle structure onto the OmpC trimer [48] showed that the gp37 tip fits well into a cavity formed at the center where the three β-barrel structures of the OmpC trimer meet [44] (S3 Movie). The amino acid residues lining the bottom surface of the LTP tip, some of which are also part of the glucose binding site, interact with the amino acid residues exposed in this OmpC cavity. This might occur through a combination of hydrogen bonds, hydrophobic and shape-complementary van der Waals interactions.

Another cluster of residues including I933, N937, and G938 form the second patch that is specific to the LPS receptor (S2 Movie). Residues N937 and G938 are at the upper rim of the glucose cavity whereas I933 is further up in a β-strand but linked to N937 and G938 residues that are part of a hydrophobic β-sheet core. Alanine mutations here resulted in loss of LPS interaction but retained OmpC binding. Thus, these residues can be regarded as LPS glucose-specificity determinants. This is consistent with the phenotype of a previously reported (rare) G938V LTF mutant, which exhibited altered host specificity. This mutant no longer formed plaques on *E*. *coli* but could form minute plaques on *Yersinia pseudotuberculosis* [42], probably because the mutation switched the LTF specificity from *E*. *coli* LPS to *Y*. *pseudotuberculosis* LPS. Surprisingly, however, the I933A, N937A, and G938A mutants bound more efficiently to OmpC than the WT LTF, even though they were unable to form plaques on OmpC^+^ *E*. *coli* K12 bacteria. This might be because the tip bound tightly to OmpC compromised its ability for dynamic interactions that require binding to both LPS and OmpC receptors, as evident from our *in vitro* binding assays. Such dynamic interactions might be essential for movement of phage on the bacterium (see below).

The third patch of residues consisting of K945, I951, and Y953 are located above the upper rim of the glucose cavity, positioned towards the side of the tip (S2 Movie). This patch is in an appropriate position to interact with residues lining the OmpC trimer cavity. Consistent with this hypothesis, mutation of any of these residues resulted in complete loss of binding to OmpC. This patch, thus, might represent an OmpC specificity determinant. However, these mutants formed plaques on *E*. *coli* K12, which normally requires OmpC interaction because the terminal glucose residues of K12 LPS are modified and inaccessible. It appears, therefore, that alanine substitutions at this site either removed clashes that otherwise interfered with K12 LPS-glucose interaction or increased the binding affinity of LTF to LPS. This pattern of gain-of-function accompanying a loss-of-function by mutation of LPS or OmpC specific patches further support the importance of dynamic interactions of LTF with the host receptors.

The tips of phage tail fibers are reported to be rich in glycines [37, 49, 50]. These glycines might be necessary to maintain the unusual conformations found in the tail fiber structures. Our mutagenesis data show that many of these glycines are essential and few, if any, substitutions were tolerated at some of these glycines. However, a notable feature of the LTF receptor-binding module is that it shows clustering of twelve glycines (four from each gp37 subunit) as part of the binding surface of the tip itself. Attempts to change, insert, or duplicate any of these glycines resulted in lethality. This is a remarkable result, and such high concentration of glycines in the binding region is unique. A strict disposition of the receptor-interacting surface therefore appears to be essential, since addition of even an extra methyl group or a peptide bond probably disrupted the arrangement and/or introduced clashes as LTF approached LPS. Glycines might also be important for proper folding of the tip and for its backbone structure since glycine has a wider range of allowed phi/psi angles [51, 52], and thus can take on conformations that are not allowed for any other residues.

### LTF tip might act as a molecular pivot

The six LTFs symmetrically attached to the baseplate of T4 phage tail represent an extraordinary device to capture the bacterial host and move on its surface for efficient infection. Since each LTF is about 145 nm long and can oscillate freely, the LTFs cover a large volume of space in their search for the host. Their primary receptor, LPS, is well-exposed and abundantly present on *E*. *coli* surface, around a million molecules per cell. Hence, a collision between an LPS-glucose and one of the eighteen glucose-binding cavities of phage LTFs is relatively frequent when a T4 phage encounters an *E*. *coli* bacterium, leading to attachment of one of the LTFs to the bacterial surface [26]. This would increase the probability of attachment of additional LTFs of the same phage to the same bacterium.

The symmetrical arrangement of receptor-binding patches around the LTF tip is well-suited for movement of phage on the bacterium. Each patch by itself probably has weak interaction with the receptor, hence it would be in a dynamic association-dissociation equilibrium. Since there are nine patches per tip, dissociation from one patch would be compensated by association with another patch(es). This, thus, creates movement of tip in the receptor cavity, changing the angle between the fiber and the bacterium, or causing rotation around the tip axis, depending on which patches associate or dissociate. Thus, each LTF tip might be acting as a molecular pivot, generating up and down as well as rotational movements. The flexible nature of LPS molecules would further contribute to these dynamic pivot movements. Similarly, the trimeric OmpC cavity aligned with the trimeric tip also provides opportunities for the LTF to pivot. Indeed, the tip docks into OmpC cavity at different angles depending on which patches are engaged (S3 Movie).

Such pivot movements must be essential for LTF function because they will allow for adjustment of the angle of each LTF to the curvature of the *E*. *coli* outer membrane as well as to movements occurring at a distance with other LTF pivots [26, 28]. It would also allow for detachment of weakly interacting tip(s) if they are not at an appropriate angle, while at the same time not allowing the phage to detach as the phage would remain anchored through the other LTFs. Our mutational analyses implicate such dynamic movements and their impairment leads to lethality, as in the case of the N937A mutation, which apparently created a stiff, inflexible OmpC binding tip. Failing to pivot, this mutant phage failed to infect even the OmpC^+^ *E*. *coli*.

We speculate that the LTF pivot movements might endow the phage with the capability to “walk” on the bacterium to search for an appropriate site for infection. It has been reported that the poles of the *E*. *coli* bacterium are enriched for phage infection sites [28]. The pivot movements would also allow the phage to attain an optimal position for infection. For instance, symmetrical anchoring of three or more LTFs (at least three LTFs were found to be essential for efficient infection [40]) might generate a signal, perhaps a tug on the baseplate, which triggers conformational changes in the baseplate proteins destabilizing the meta-stable hexagonal baseplate, and unpinning and downward rotation of the short tail fibers. The tips of the short tail fibers then irreversibly bind to LPS receptors that then triggers tail sheath contraction, penetration of the tail tube into *E*. *coli*, and flow of phage genome into *E*. *coli* cell.

Thus, phage T4 appears to have evolved a sophisticated pivoting device to maximize its infection efficiency. Though this device is costly, requiring production of about ten different proteins, it imparts enormous advantage to T4 over its competitors to efficiently capture its nutrient source, the host bacterium, at a faster time scale. Although mechanisms might vary, this concept of “capture and search” might be common in the phage world and even in eukaryotic viruses, as it allows viruses to scan a large surface area of the host and efficiently engage with the sites of infection. In future, it should be possible to design pivots with altered or broadened host specificity and target these engineered phages to eliminate multi-drug resistant bacterial infections [3].

## Materials and methods

### Ethics statement

Animal experiments were conducted in accordance with the recommendations in the Guide for the Care and Use of Laboratory Animals of the National Institutes of Health. The protocols were reviewed and approved by the Institutional Animal Care and Use Committees of The Catholic University of America (Office of Laboratory Animal Welfare assurance number: A4431-01).

### Bacteria and plasmids

*Escherichia coli* B40 (sup^+^), BL21 (DE3) (Tyr sup^+^), BL21 (DE3) (Gly sup^+^), and BL21 (DE3) (Arg sup^+^) were used as amber suppressors for initial crosses and preparation of phage stocks. The sup^-^ *E*. *coli* P301 (B strain) and sup^-^ *E*. *coli* D21 (K12 strain) (*E*. *coli* Genomic Resource Center, Yale University, New Haven, CT) were used in marker rescue assays. *E*. *coli* BL21 (sup^-^) was used as the host for pET28b plasmid carrying *g37* alanine substitution mutants and mutant libraries. The *E*. *coli* XL10-Gold cells (Agilent Technologies, Santa Clara, CA) or DH5α cells (New England BioLabs, Ipswich, MA), were used for initial transformation and maintenance of recombinant constructs. For protein expression, sequence-confirmed recombinant clones were transferred into the expression strain *E*. *coli* BL21 (DE3) (Agilent Technologies, Santa Clara, CA) or *E*. *coli* BL21 (DE3) pLysS (Stratagene, La Jolla, CA) to allow IPTG (isopropyl-β-D-thiogalactopyranoside)-induced overexpression of recombinant proteins [53]. *E*. *coli* BZB1109 (a gift from Prof. Tillman Schirmer, Biozentrum, University of Basel, Basel, Switzerland) was used to express OmpC. The T7 expression plasmids pET28b, pCDFDuet-1, pET21a, and pET30a (EMD Millipore, MA), were used as the cloning vectors. Purified phage T4 genomic DNA was used as a template to amplify T4 *g37* and its mutants.

### Construction of gp37 expression plasmids

The T4 *g38* and *g57* genes were amplified by PCR using T4 genomic DNA as template and specific end primers containing NdeI and XhoI restriction sites for directional cloning. The *g38* DNA was cloned into the multiple cloning site 2 (MCS2) of the pCDF Duet-1 vector and *g57A* was cloned into the pET30a and pET21a vectors.

All the amber mutations, alanine substitution mutants, and mutant libraries by combinatorial mutagenesis were introduced into T4 *g37* by splicing by overlap extension (SOE) strategy [54–57]. Four oligonucleotides (primer 1: 5’-forward, primer 2: mutant reverse, primer 3: mutant forward, and primer 4: 3’-reverse) and three successive PCRs were used to introduce the mutations. The NheI and XhoI restriction enzyme sites were included in primers 1 and 4, respectively, for directional cloning of the DNA into the pET28b vector.

For protein expression and purification, BamHI and NotI sites were included in primers 1 & 4, respectively, for directional cloning into the multiple cloning site 1 (MCS1) of the pCDF Duet-1 vector which contained *g38* in the MCS2 as described by [44]. Insertion of the recombinant DNA resulted in in-frame fusion with a 13-aa vector sequence containing a hexa-histidine sequence at the N-terminus of the gp37 constructs. In order to get efficient recombination and transfer of amber mutations or of mutant libraries into T4 genome, the coding sequences of *g37* (amino acids 344–1026) and the flanking noncoding and *g38* (amino acids 1–101) sequences were included. All gp37 recombinant proteins included the amino acids 799 to 1026. The amplified DNAs were purified by agarose gel electrophoresis, digested with respective restrictions enzymes and ligated with the gel-purified vector that was also digested with the same restriction enzymes. The ligated DNA was then transformed into *E*. *coli* XL10-Gold cells, and plasmid DNAs were prepared from individual transformants by the alkaline lysis method using a Miniprep kit (Thermo Fisher Scientific, Waltham, MA). The entire insert was sequenced (Retrogen Inc., San Diego, CA) to confirm that there were no errors in the cloned DNA. The pCDF Duet-1 plasmid containing the *g37* and *g38* constructs and the pET30a plasmid containing *g57* were co-transformed into *E*. *coli* BL21 (DE3) pLysS for overexpression of the recombinant gp37 [44]. The pET28b plasmids carrying the *g37* alanine mutants and mutant libraries were transformed into *E*. *coli* BL21 (sup^-^). The pET28b plasmids carrying the *g37* amber mutants were transformed into their respective sup^+^ *E*. *coli* strains.

### Purification of recombinant gp37 trimers

Expression and purification of recombinant gp37 was carried out as described by Bartual et al., 2006 [44]. The *E*. *coli* strain BL21 (DE3) pLysS harboring pCDF Duet-1 *g37*, *g38*, and pET30a *g57* was grown in Moore’s media supplemented with streptomycin (50 mg/L), kanamycin (35 mg/L) and chloramphenicol (35 mg/L). Expression was induced with 1 mM IPTG at 20°C overnight (16 hr). Cells were harvested by centrifugation at 4,000 *g* for 12 min at 4°C, and stored at -80°C until use. The pellets were resuspended in 40 ml per liter culture of HisTrap binding buffer (50 mM Tris-HCl, pH 8, 300 mM NaCl, and 10 mM imidazole) containing protease inhibitor cocktail (Roche Diagnostics, IN) and lysed by French press (Amicon). His-tagged soluble proteins were separated from the cell debris by centrifugation at 34,000 *g* at 4°C for 25 min and loaded onto a 1 ml HisTrap column (AKTA Prime, GE Healthcare) pre-equilibrated with the binding buffer. Unbound proteins were washed away by the same buffer supplemented with 50 mM imidazole. The bound proteins were eluted with 50–500 mM linear imidazole gradient in the same buffer containing 100 mM NaCl. The proteins were further purified by size-exclusion chromatography using Hi-Load 16/60 Superdex-200 (prep-grade) column (FPLC, GE Healthcare) in phosphate-buffered saline (PBS; pH 7.4). The trimer peak fractions corresponding to about 80 kDa were pooled, concentrated by Amicon membrane filtration, and stored at -80ºC.

### Purification of recombinant OmpC trimers

Expression and purification of OmpC was carried out as described by Basle et al., [43]. Briefly, 1 ml overnight grown *E*. *coli* strain BZB1109 was inoculated into 1 L of Luria broth (LB) supplemented with ampicillin (50 mg/L) and kanamycin (35 mg/L) and incubated at 37ºC for 8 hr. Cells were harvested by centrifugation at 4,000 *g* for 12 min at 4°C, and stored at -80°C until use. The pellets were resuspended in 40 ml of resuspension buffer (20 mM Tris-HCl, pH 8) containing 1 tablet of protease inhibitor cocktail and lysed by French press. SDS was added to a final concentration of 2% and samples were incubated at 37°C for 1 hr followed by centrifugation at 140,000 *g*. The pellet was then pre-extracted with 0.125% octyl-POE detergent containing 20 mM NaH_2_PO_4_ (pH 7.3) buffer followed by solubilization with 3% octyl-POE detergent and centrifugation at 140,000 x *g*. The soluble protein was then dialyzed against PBE chromatofocusing binding buffer (25 mM histidine-HCl, 5 mM EDTA, 1% octyl-POE, pH 6) and loaded onto the MonoP 5/200 GL chromatofocusing column (GE Healthcare). The protein was eluted with PBE-74 buffer [12.5% polybuffer74 (GE Healthcare), 25 mM Histidine-HCl, 5mM EDTA, 1% octyl-POE, pH 3]. The eluted fraction from PBE chromatofocusing was then purified by size-exclusion chromatography using Hi-Load 16/60 Superdex-200 (prep-grade) column (FPLC, GE Healthcare) in PBS containing 0.6% octyl POE. The OmpC trimer fractions were pooled, concentrated, and stored at -80ºC.

### Production of OmpC-specific polyclonal antibodies

Six to eight week old female Balb/c mice (19–21 g) were purchased from Jackson Laboratories (Bar Harbor, Maine), randomly grouped and acclimated for 7 days. Eight mice were immunized with the purified OmpC trimers (50 μg/mouse) adsorbed on Alhydrogel (Brenntag Biosector, Denmark) containing 0.19 mg of aluminum per dose. Mice were immunized via the intramuscular route on day 0, followed by 2 boosts on days 30 and 60. Blood was collected form the tail vein at day 0 (pre-immunized), days 28, and 44, and the terminal blood was collected 14 days after the final boost prior to euthanasia. Blood samples were left at room temperature for 45 min to allow clotting. Sera were then separated by centrifugation at 2,000 *g* for 10 min and stored at -80ºC. Anti-OmpC antibody titers were determined by ELISA, by coating the wells of a flat-bottomed 96-well plate (MaxiSorp, Nunc, Thermo Scientific, Rochester, NY) with OmpC antigen (100 ng/well) for overnight at 4°C. After two washes with PBS containing 0.05% Tween 20 (PBS-T), wells were blocked with 1X casein blocking buffer (Sigma Aldrich, USA) for 2 hr at room temperature. Fivefold serially diluted mice sera were added to the wells and incubated at 37°C for 2 hr. After four washes with PBS-T, the wells were incubated with 1:5,000 diluted HRP-conjugated rabbit anti-mouse IgG (Invitrogen, Camarillo, CA) at 37°C for 1 hr. After washing, the TMB (3,3’,5,5’-tetramethylbenzidine) substrate was added and incubated for 25 min to develop the color. The absorbance at 650 nm was determined using an ELISA reader (VERSA max, Molecular Devices) (S2 Fig).

### Introduction of amber mutations into T4 genome

A series of amber stop codons were introduced into *g37* and amber phages were isolated. Initially, the Y932am phage was isolated. To obtain this phage, the Y932am mutant plasmid was constructed and transformed into the BL21 (DE3) (Tyr sup^+^) *E*. *coli* strain. A single colony was grown in LB-M9CA media to obtain about 2 x 10^8^ cells/ml, which were then infected with N52am (Q395am) phage at the multiplicity of infection of 1 at 37ºC for 40 min. Unabsorbed phages were removed by centrifugation at 5,000 x *g* for 10 min. Progeny phages were released from the *E*. *coli* cells by adding chloroform and DNase I and incubating at 37ºC for 30 min. The cell debris was separated by another slow speed centrifugation at 5,000 x *g* for 10 min. The phages were first plated on permissive *E*. *coli* Tyr sup^+^ strain and individual plaque was then screened on *E*. *coli* P301 (sup^-^) and 13 different sup^+^ *E*. *coli* strains [46] to isolate the desired amber phage. All the glycine amber phages (G829am, G853am, G913am, G921am, G938am, G943am, G964am, and G978am) were isolated by infecting the *E*. *coli* BL21 (DE3) Gly sup^+^ strain containing their respective amber plasmid with the Y932am phage. The rest of the amber phages (S868am, S924am, R870am, and R954am) were isolated by infecting the respective sup^+^ *E*. *coli* strains containing the amber plasmid with the G938am phage. The individual plaques were then screened on P301 *E*. *coli* (sup^-^) and respective sup^+^ *E*. *coli* lawns to isolate the desired amber phages. Insertion of the amber mutation was confirmed by DNA sequencing of PCR amplified *g37* fragment from individual mutant plaques. The phenotypes of amber phages were tested on 13 different amino acid sup^+^ *E*. *coli* B (*OmpC*^*-*^) strains [46] available in our laboratory.

### Transfer of combinatorial mutations and alanine mutations into phage T4 genome

Construction of mutant libraries by combinatorial mutagenesis and transfer into T4 genome was carried out as described previously [57] (S3 Fig). Briefly, a 5 μl aliquot of each mutant culture (~10^5^ *E*. *coli* cells carrying the mutant plasmid) was spotted in duplicate on a plate spread with *E*. *coli* P301 (sup^-^). An aliquot (~10^5^ phages) of either Y932am or N52am (Q395am) phage was then spotted on top of the mutant spot. The N52am phage was used as a control to ascertain that the transformants contained the *g37* insert. Every insert-containing transformant should show lysis with the N52am phage since it is far from the amber mutation site (positive marker rescue). On the other hand, since Y932am is very close to the mutation site (within 30 bp from the amber site), most recombinational exchanges involving this amber site would result in reciprocal exchange of the mutagenized sequence into the phage genome. Each transformant was then phenotypically scored as functional or null depending on the appearance of plaques. For each library, a set of null or functional phenotypes were selected and the corresponding mutant plasmid DNA was sequenced. Transfer of alanine substitutions into the T4 genome was performed similarly by spotting the mutant plasmid-containing *E*. *coli* BL21 (sup^-^) on a plate spread with *E*. *coli* P301 (sup^-^), followed by spotting a double amber (S924amR954am) mutant phage on each spot.

### UV crosslinking of gp37-LPS complex

Purified gp37 trimers were labelled with amine-reactive diazirine crosslinkers (NHS-LC-Diazirine) (Thermo Scientific, Rockford, IL) by adding 50-fold molar excess of the reagent to gp37 and incubated for 2 hr on ice. The reaction was stopped by adding quenching buffer (100 mM Tris-HCl, pH 8). Unreacted crosslinker was removed by passing through the Zeba Spin Desalting column (Thermo Scientific, Rockford, IL). The diazirine-labelled gp37 (150 μg) was then incubated with 500 μg of LPS (*E*. *coli* 0111:B4; Sigma-Aldrich, St Louis, MO) in a 200 μl reaction volume at 37°C for 1 hr. As controls, the same amounts of diazirine-labelled gp37 and LPS were incubated in the same buffer in separate tubes. The reaction mixtures were UV irradiated for 15 min using Stratagene Stratalinker 2400. Crosslinked products were then separated by size-exclusion column chromatography (Superose 6 10/300 GL, GE Healthcare Bioscience). The fractions were analyzed by SDS-PAGE and stained with silver stain. A step-by-step protocol can be found at http://dx.doi.org/10.17504/protocols.io.77nhrme.

### Formation of gp37-OmpC complex

Wells of a flat-bottomed 96-well microtiter plate were coated in duplicate with WT gp37 or alanine mutant trimers (100 ng/well) for overnight at 4°C. After two washes with PBS-T, wells were blocked with 1X casein blocking buffer (Sigma Aldrich, USA) for 2 hr at room temperature. OmpC was added to the wells at a gp37:OmpC molar ratio of 1:1 and incubated at 37°C for 2 hr. After washing 4 times with PBS-T, anti-OmpC mouse sera at a dilution of 1:5,000 was added and the plates were incubated for 1 hr at 37ºC followed by 4 times washing and incubation with 1: 5,000 diluted HRP-conjugated rabbit anti-mouse IgG (Invitrogen, Camarillo, CA) at 37°C for 1 hr. After washing 4 times, the TMB (3,3’,5,5’-tetramethylbenzidine) substrate was added and incubated for 25 min to develop the color. The absorbance at 650 nm was determined using an ELISA reader (VERSA max, Molecular Devices). Concentration of bound OmpC was determined from a standard curve constructed for each plate with serially diluted known concentrations of OmpC incubated with anti-OmpC antibody. The cutoff point (6,867 pg) was calculated by mean + 3 standard deviation (SD) of the negative values (those mutations that were not tolerated in the marker rescue assay and showed very poor binding to OmpC). A step-by-step protocol can be found at http://dx.doi.org/10.17504/protocols.io.77vhrn6.

### Binding of gp37 trimers to *E*. *coli*

About 10^8^ *E*. *coli* cells were centrifuged at 2,300 *g* for 3 min at 4°C in a low-bind Eppendorf tube. The cells were resuspended in PBS and purified WT gp37 and alanine mutant trimers were added to about 3 x 10^5^ gp37 molecules per cell in a reaction volume of 150 μl. Samples were incubated at 37ºC for 2 hr, and the *E*. *coli* cells were sedimented by centrifugation at 2,300 *g* for 3 min at 4°C, followed by washing twice with 1 ml of PBS. The pellet was resuspended in 10 μl of PBS, transferred to a new tube, and 10 μl of 2X SDS sample buffer was added. The proteins were separated by electrophoresis, transferred to a polyvinylidene difluoride (PVDF) membrane, and incubated with monoclonal anti-histag antibodies (Thermo Fisher Scientific, Waltham, MA) to detect the bound his-tagged gp37 proteins. HRP-conjugated rabbit anti-mouse IgG (Invitrogen, Camarillo, CA) was used as the second antibody, and the bands were visualized by adding a chromogenic substrate (Invitrogen) or exposing to an X-ray film after adding the ECL reagent (Pierce Biotechnology, Rockford, IL). Each experiment included two negative controls: (i) gp37 was omitted, which showed several background bands due to nonspecific interactions, and (ii) *E*. *coli* cells were omitted, which showed no bands. A step-by-step protocol can be found at http://dx.doi.org/10.17504/protocols.io.77xhrpn.

### Structural analysis

Structural analyses of T4 gp37 (PDB ID: 2XGF) were performed using PYMOL (PyMOL Molecular Graphics System, Schrödinger, LLC).

### Statistical analysis

Results are expressed as mean ± SD. Statistical comparisons between gp37 WT and mutants were evaluated by ANOVA. A value of P < 0.05 was considered to indicate statistical significance.

## Supporting information

S1 Fig Schematic representation of LPS structures. The structure of *E*. *coli* B type LPS was generated based on the structure described by Washizaki et al. [21]. The LPS structure of *E*. *coli* D21 (K12) was adopted from the structure described by Pulido et al. [19]. Abbreviations used in the figure are: Glu, Glucose; Gal, Galactose; NAG, N-acetylglucosamine; KDO, 3-deoxy-D-mano-oct-2-ulosonic acid; Hep, L-glycerol-D-mano heptose; P, Phosphate; OM, outer membrane; OmpC, outer membrane protein C.
(TIF)

S2 Fig Titration of anti-OmpC antibodies. Anti-OmpC antibodies were produced by immunizing mice with the purified OmpC trimer. The endpoint titer was determined by applying serially diluted immune sera into the wells of a 96-well ELISA plate coated with purified OmpC.
(TIF)

S3 Fig Combinatorial mutant library construction and analysis. (A, B) Schematics showing the construction of libraries by introducing all possible codons at a given amino acid and transferring each mutation into phage genome by recombination using an amber mutation (e.g., Y932am) at a nearby flanking amino acid. The phenotype, functional or null, of the resultant progeny phage was determined by their ability to form plaques on an appropriate *E*. *coli* strain. The mutation giving rise to a phenotype is determined by PCR amplification of mutant DNA and DNA sequencing.
(TIF)

S1 Movie Patches of LPS and OmpC receptor specificity around the ball of the LTF tip. (MPG)

S2 Movie Glucose binding cavity of the LTF tip. (MPG)

S3 Movie Docking of LTF tip into the OmpC trimer cavity at different angles. (MPG)

## References

[1] Hendrix RW. Bacteriophage genomics. Curr Opin Microbiol. 2003;6(5):506–11. Epub 2003/10/24. 10.1016/j.mib.2003.09.004 . 14572544

[2] Hatfull GF, Hendrix RW. Bacteriophages and their genomes. Curr Opin Virol. 2011;1(4):298–303. Epub 2011/10/29. 10.1016/j.coviro.2011.06.009 22034588 PMC3199584

[3] Tao P, Wu X, Tang WC, Zhu J, Rao V. Engineering of Bacteriophage T4 Genome Using CRISPR-Cas9. ACS Synth Biol. 2017;6(10):1952–61. 10.1021/acssynbio.7b00179 28657724 PMC5771229

[4] Segall AM, Roach DR, Strathdee SA. Stronger together? Perspectives on phage-antibiotic synergy in clinical applications of phage therapy. Curr Opin Microbiol. 2019;51:46–50. Epub 2019/06/22. 10.1016/j.mib.2019.03.005 . 31226502

[5] Maniloff J, Ackermann HW. Taxonomy of bacterial viruses: establishment of tailed virus genera and the order Caudovirales. Arch Virol. 1998;143(10):2051–63. Epub 1998/12/18. 10.1007/s007050050442 . 9856093

[6] Miller ES, Kutter E, Mosig G, Arisaka F, Kunisawa T, Ruger W. Bacteriophage T4 genome. Microbiol Mol Biol Rev. 2003;67(1):86–156, table of contents. Epub 2003/03/11. 10.1128/MMBR.67.1.86-156.2003 12626685 PMC150520

[7] Yap ML, Rossmann MG. Structure and function of bacteriophage T4. Future Microbiol. 2014;9(12):1319–27. Epub 2014/12/18. 10.2217/fmb.14.91 25517898 PMC4275845

[8] Arisaka F, Engel J, Klump H. Contraction and dissociation of the bacteriophage T4 tail sheath induced by heat and urea. Prog Clin Biol Res. 1981;64:365–79. Epub 1981/01/01. . 7330053

[9] Kanamaru S, Leiman PG, Kostyuchenko VA, Chipman PR, Mesyanzhinov VV, Arisaka F, et al Structure of the cell-puncturing device of bacteriophage T4. Nature. 2002;415(6871):553–7. Epub 2002/02/02. 10.1038/415553a . 11823865

[10] Leiman PG, Arisaka F, van Raaij MJ, Kostyuchenko VA, Aksyuk AA, Kanamaru S, et al Morphogenesis of the T4 tail and tail fibers. Virol J. 2010;7:355 Epub 2010/12/07. 10.1186/1743-422X-7-355 21129200 PMC3004832

[11] Taylor NMI, van Raaij MJ, Leiman PG. Contractile injection systems of bacteriophages and related systems. Mol Microbiol. 2018;108(1):6–15. Epub 2018/02/07. 10.1111/mmi.13921 . 29405518

[12] Leiman PG, Kanamaru S, Mesyanzhinov VV, Arisaka F, Rossmann MG. Structure and morphogenesis of bacteriophage T4. Cell Mol Life Sci. 2003;60(11):2356–70. Epub 2003/11/20. 10.1007/s00018-003-3072-1 . 14625682 PMC11138918

[13] Mathews CK, Kutter E., Mosig G., and Berget P.B. Bacteriophage T4. Washington, D.C: American Society for Microbiology; 1983.

[14] King J, Laemmli UK. Polypeptides of the tail fibres of bacteriophage T4. J Mol Biol. 1971;62(3):465–77. Epub 1971/12/28. 10.1016/0022-2836(71)90148-3 . 5136579

[15] Granell M, Namura M, Alvira S, Kanamaru S, van Raaij MJ. Crystal Structure of the Carboxy-Terminal Region of the Bacteriophage T4 Proximal Long Tail Fiber Protein Gp34. Viruses. 2017;9(7). Epub 2017/07/01. 10.3390/v9070168 28665339 PMC5537660

[16] Cerritelli ME, Wall JS, Simon MN, Conway JF, Steven AC. Stoichiometry and domainal organization of the long tail-fiber of bacteriophage T4: a hinged viral adhesin. J Mol Biol. 1996;260(5):767–80. Epub 1996/08/02. 10.1006/jmbi.1996.0436 . 8709154

[17] Hashemolhosseini S, Stierhof YD, Hindennach I, Henning U. Characterization of the helper proteins for the assembly of tail fibers of coliphages T4 and lambda. J Bacteriol. 1996;178(21):6258–65. Epub 1996/11/01. 10.1128/jb.178.21.6258-6265.1996 8892827 PMC178498

[18] Bartual SG, Otero JM, Garcia-Doval C, Llamas-Saiz AL, Kahn R, Fox GC, et al Structure of the bacteriophage T4 long tail fiber receptor-binding tip. Proc Natl Acad Sci U S A. 2010;107(47):20287–92. Epub 2010/11/03. 10.1073/pnas.1011218107 21041684 PMC2996694

[19] Pulido D, Moussaoui M, Andreu D, Nogues MV, Torrent M, Boix E. Antimicrobial action and cell agglutination by the eosinophil cationic protein are modulated by the cell wall lipopolysaccharide structure. Antimicrob Agents Chemother. 2012;56(5):2378–85. Epub 2012/02/15. 10.1128/AAC.06107-11 22330910 PMC3346588

[20] Dawes J. Characterisation of the bacteriophage T4 receptor site. Nature. 1975;256(5513):127–8. Epub 1975/07/10. 10.1038/256127a0 . 1097935

[21] Washizaki A, Yonesaki T, Otsuka Y. Characterization of the interactions between Escherichia coli receptors, LPS and OmpC, and bacteriophage T4 long tail fibers. Microbiologyopen. 2016;5(6):1003–15. Epub 2016/06/09. 10.1002/mbo3.384 27273222 PMC5221442

[22] Montag D, Hashemolhosseini S, Henning U. Receptor-recognizing proteins of T-even type bacteriophages. The receptor-recognizing area of proteins 37 of phages T4 TuIa and TuIb. J Mol Biol. 1990;216(2):327–34. Epub 1990/11/20. 10.1016/S0022-2836(05)80324-9 . 2147721

[23] Prehm P, Jann B, Jann K, Schmidt G, Stirm S. On a bacteriophage T3 and T4 receptor region within the cell wall lipopolysaccharide of Escherichia coli B. J Mol Biol. 1976;101(2):277–81. Epub 1976/02/25. 10.1016/0022-2836(76)90377-6 . 772219

[24] Mutoh N, Furukawa H, Mizushima S. Role of lipopolysaccharide and outer membrane protein of Escherichia coli K-12 in the receptor activity for bacteriophage T4. J Bacteriol. 1978;136(2):693–9. Epub 1978/11/01. 361717 10.1128/jb.136.2.693-699.1978 PMC218595

[25] Yu F, Mizushima S. Roles of lipopolysaccharide and outer membrane protein OmpC of Escherichia coli K-12 in the receptor function for bacteriophage T4. J Bacteriol. 1982;151(2):718–22. Epub 1982/08/01. 7047495 10.1128/jb.151.2.718-722.1982 PMC220313

[26] Hu B, Margolin W, Molineux IJ, Liu J. Structural remodeling of bacteriophage T4 and host membranes during infection initiation. Proc Natl Acad Sci U S A. 2015;112(35):E4919–28. Epub 2015/08/19. 10.1073/pnas.1501064112 26283379 PMC4568249

[27] Hu B, Margolin W, Molineux IJ, Liu J. The bacteriophage t7 virion undergoes extensive structural remodeling during infection. Science. 2013;339(6119):576–9. Epub 2013/01/12. 10.1126/science.1231887 23306440 PMC3873743

[28] Edgar R, Rokney A, Feeney M, Semsey S, Kessel M, Goldberg MB, et al Bacteriophage infection is targeted to cellular poles. Mol Microbiol. 2008;68(5):1107–16. Epub 2008/03/28. 10.1111/j.1365-2958.2008.06205.x 18363799 PMC3740151

[29] Rothenberg E, Sepulveda LA, Skinner SO, Zeng L, Selvin PR, Golding I. Single-virus tracking reveals a spatial receptor-dependent search mechanism. Biophys J. 2011;100(12):2875–82. Epub 2011/06/22. 10.1016/j.bpj.2011.05.014 21689520 PMC3123979

[30] Parent KN, Erb ML, Cardone G, Nguyen K, Gilcrease EB, Porcek NB, et al OmpA and OmpC are critical host factors for bacteriophage Sf6 entry in Shigella. Mol Microbiol. 2014;92(1):47–60. Epub 2014/03/29. 10.1111/mmi.12536 24673644 PMC4034267

[31] Bhardwaj A, Olia AS, Cingolani G. Architecture of viral genome-delivery molecular machines. Curr Opin Struct Biol. 2014;25:1–8. Epub 2014/06/01. 10.1016/j.sbi.2013.10.005 24878339 PMC4040186

[32] Riede I. Receptor specificity of the short tail fibres (gp12) of T-even type Escherichia coli phages. Mol Gen Genet. 1987;206(1):110–5. Epub 1987/01/01. 10.1007/bf00326544 . 3553859

[33] Kostyuchenko VA, Navruzbekov GA, Kurochkina LP, Strelkov SV, Mesyanzhinov VV, Rossmann MG. The structure of bacteriophage T4 gene product 9: the trigger for tail contraction. Structure. 1999;7(10):1213–22. Epub 1999/11/05. 10.1016/s0969-2126(00)80055-6 . 10545330

[34] Kostyuchenko VA, Chipman PR, Leiman PG, Arisaka F, Mesyanzhinov VV, Rossmann MG. The tail structure of bacteriophage T4 and its mechanism of contraction. Nat Struct Mol Biol. 2005;12(9):810–3. Epub 2005/08/24. 10.1038/nsmb975 . 16116440

[35] Taylor NM, Prokhorov NS, Guerrero-Ferreira RC, Shneider MM, Browning C, Goldie KN, et al Structure of the T4 baseplate and its function in triggering sheath contraction. Nature. 2016;533(7603):346–52. Epub 2016/05/20. 10.1038/nature17971 . 27193680

[36] Yap ML, Klose T, Arisaka F, Speir JA, Veesler D, Fokine A, et al Role of bacteriophage T4 baseplate in regulating assembly and infection. Proc Natl Acad Sci U S A. 2016;113(10):2654–9. Epub 2016/03/02. 10.1073/pnas.1601654113 26929357 PMC4791028

[37] Dunne M, Denyes JM, Arndt H, Loessner MJ, Leiman PG, Klumpp J. Salmonella Phage S16 Tail Fiber Adhesin Features a Rare Polyglycine Rich Domain for Host Recognition. Structure. 2018;26(12):1573–82 e4. Epub 2018/09/25. 10.1016/j.str.2018.07.017 . 30244968

[38] Salazar AJ, Sherekar M, Tsai J, Sacchettini JC. R pyocin tail fiber structure reveals a receptor-binding domain with a lectin fold. PLoS One. 2019;14(2):e0211432 Epub 2019/02/06. 10.1371/journal.pone.0211432 30721244 PMC6363177

[39] Arisaka F. Assemblyand infection process of bacteriophage T4. Chaos. 2005;15(4):047502 Epub 2006/01/07. 10.1063/1.2142136 . 16396595

[40] Goldberg E, Grinius L., and Letellier L. Recognition, attachment, and injection In: Karam JD, Drake J.W., Kreuzer K.N., Mosig G., Hall D.H., Eiserling F.A., Black L.W., Spicer E.K., Kutter E., Carlson K., and Miller E.S., editor. Molecular biology of bacteriophage T4. Washington, D.C: American Society for Microbiology; 1994 p. 347–56.

[41] Hashemolhosseini S, Montag D, Kramer L, Henning U. Determinants of receptor specificity of coliphages of the T4 family. A chaperone alters the host range. J Mol Biol. 1994;241(4):524–33. Epub 1994/08/26. 10.1006/jmbi.1994.1529 . 8057378

[42] Tetart F, Repoila F, Monod C, Krisch HM. Bacteriophage T4 host range is expanded by duplications of a small domain of the tail fiber adhesin. J Mol Biol. 1996;258(5):726–31. Epub 1996/05/24. 10.1006/jmbi.1996.0281 . 8637004

[43] Basle A, Rummel G, Storici P, Rosenbusch JP, Schirmer T. Crystal structure of osmoporin OmpC from E. coli at 2.0 A. J Mol Biol. 2006;362(5):933–42. Epub 2006/09/05. 10.1016/j.jmb.2006.08.002 . 16949612

[44] Bartual SG, Garcia-Doval C, Alonso J, Schoehn G, van Raaij MJ. Two-chaperone assisted soluble expression and purification of the bacteriophage T4 long tail fibre protein gp37. Protein Expr Purif. 2010;70(1):116–21. Epub 2009/11/17. 10.1016/j.pep.2009.11.005 . 19913618

[45] Rezania S, Amirmozaffari N, Tabarraei B, Jeddi-Tehrani M, Zarei O, Alizadeh R, et al Extraction, Purification and Characterization of Lipopolysaccharide from Escherichia coli and Salmonella typhi. Avicenna J Med Biotechnol. 2011;3(1):3–9. Epub 2011/01/01. 23407691 PMC3558174

[46] Normanly J, Kleina LG, Masson JM, Abelson J, Miller JH. Construction of Escherichia coli amber suppressor tRNA genes. III. Determination of tRNA specificity. J Mol Biol. 1990;213(4):719–26. Epub 1990/06/20. 10.1016/S0022-2836(05)80258-X . 2141650

[47] Trott O, Olson AJ. AutoDock Vina: improving the speed and accuracy of docking with a new scoring function, efficient optimization, and multithreading. J Comput Chem. 2010;31(2):455–61. Epub 2009/06/06. 10.1002/jcc.21334 19499576 PMC3041641

[48] Schneidman-Duhovny D, Inbar Y, Nussinov R, Wolfson HJ. PatchDock and SymmDock: servers for rigid and symmetric docking. Nucleic Acids Res. 2005;33(Web Server issue):W363–7. Epub 2005/06/28. 10.1093/nar/gki481 15980490 PMC1160241

[49] Trojet SN, Caumont-Sarcos A, Perrody E, Comeau AM, Krisch HM. The gp38 adhesins of the T4 superfamily: a complex modular determinant of the phage's host specificity. Genome Biol Evol. 2011;3:674–86. Epub 2011/07/13. 10.1093/gbe/evr059 21746838 PMC3157838

[50] Thomassen E, Gielen G, Schutz M, Schoehn G, Abrahams JP, Miller S, et al The structure of the receptor-binding domain of the bacteriophage T4 short tail fibre reveals a knitted trimeric metal-binding fold. J Mol Biol. 2003;331(2):361–73. Epub 2003/07/31. 10.1016/s0022-2836(03)00755-1 . 12888344

[51] Richardson JS. The anatomy and taxonomy of protein structure. Adv Protein Chem. 1981;34:167–339. Epub 1981/01/01. 10.1016/s0065-3233(08)60520-3 . 7020376

[52] Ramachandran GN, Ramakrishnan C, Sasisekharan V. Stereochemistry of polypeptide chain configurations. J Mol Biol. 1963;7:95–9. Epub 1963/07/01. 10.1016/s0022-2836(63)80023-6 . 13990617

[53] Tolia NH, Joshua-Tor L. Strategies for protein coexpression in Escherichia coli. Nat Methods. 2006;3(1):55–64. Epub 2005/12/22. 10.1038/nmeth0106-55 . 16369554

[54] Studier FW, Rosenberg AH, Dunn JJ, Dubendorff JW. Use of T7 RNA polymerase to direct expression of cloned genes. Methods Enzymol. 1990;185:60–89. Epub 1990/01/01. 10.1016/0076-6879(90)85008-c . 2199796

[55] Horton RM, Hunt HD, Ho SN, Pullen JK, Pease LR. Engineering hybrid genes without the use of restriction enzymes: gene splicing by overlap extension. Gene. 1989;77(1):61–8. Epub 1989/04/15. 10.1016/0378-1119(89)90359-4 . 2744488

[56] Sathaliyawala T, Islam MZ, Li Q, Fokine A, Rossmann MG, Rao VB. Functional analysis of the highly antigenic outer capsid protein, Hoc, a virus decoration protein from T4-like bacteriophages. Mol Microbiol. 2010;77(2):444–55. Epub 2010/05/26. 10.1111/j.1365-2958.2010.07219.x 20497329 PMC2909354

[57] Rao VB, Mitchell MS. The N-terminal ATPase site in the large terminase protein gp17 is critically required for DNA packaging in bacteriophage T4. J Mol Biol. 2001;314(3):401–11. Epub 2002/02/16. 10.1006/jmbi.2001.5169 . 11846554

