# Supplementary figures and images for: Molecular anatomy of the receptor binding module of a bacteriophage long tail fiber
Source: PLoS Pathog. 2019 Dec 19;15(12):e1008193. doi: 10.1371/journal.ppat.1008193 (PMC6957217; doi:10.1371/journal.ppat.1008193)

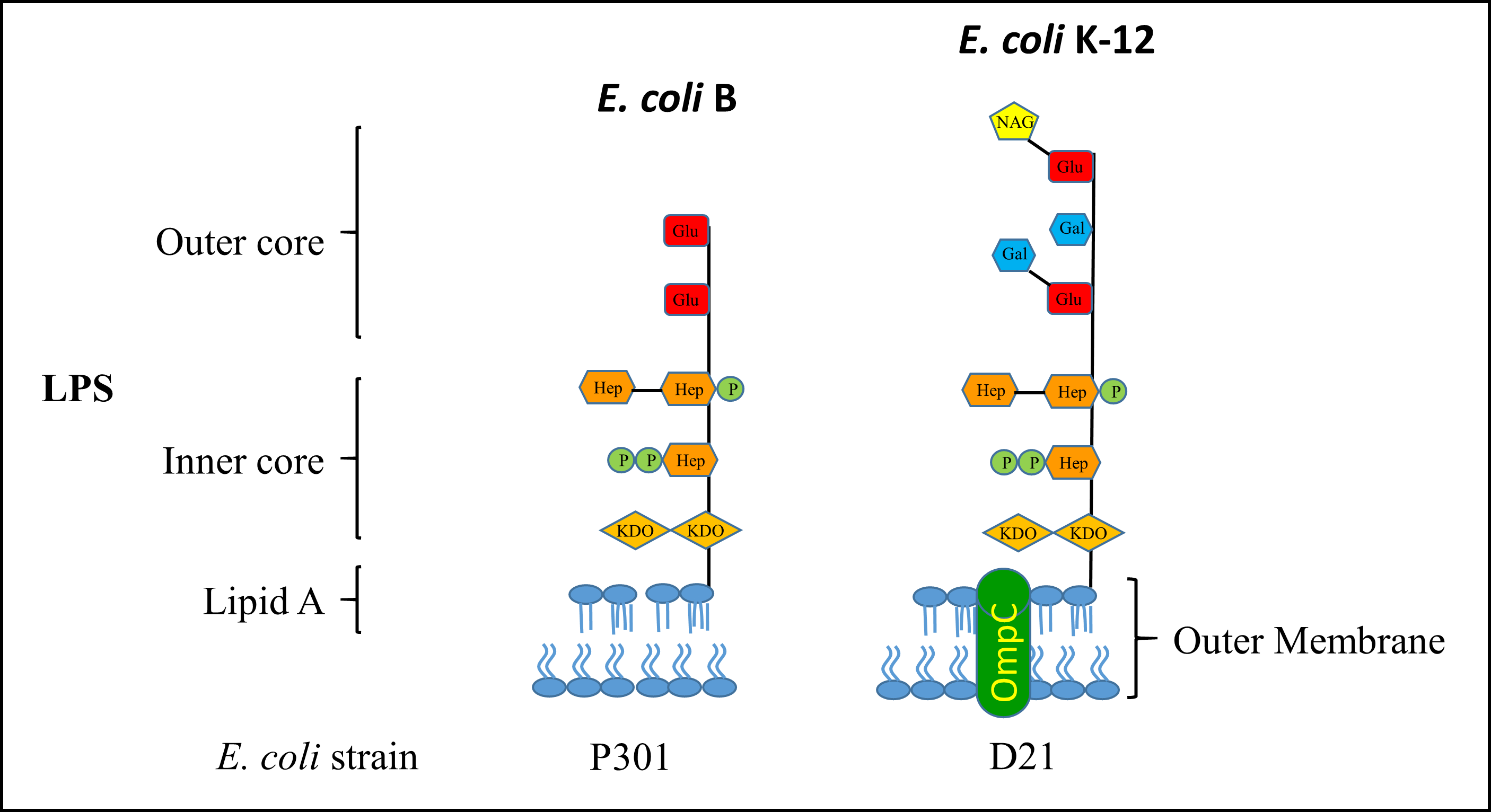

Supplement: S1 Fig — The structure of E. coli B type LPS was generated based on the structure described by Washizaki et al. [21]. The LPS structure of E. coli D21 (K12) was adopted from the structure described by Pulido et al. [19]. Abbreviations used in the figure are: Glu, Glucose; Gal, Galactose; NAG, N-acetylglucosamine; KDO, 3-deoxy-D-mano-oct-2-ulosonic acid; Hep, L-glycerol-D-mano heptose; P, Phosphate; OM, outer membrane; OmpC, outer membrane protein C. (TIF) [file ppat.1008193.s001.tif]

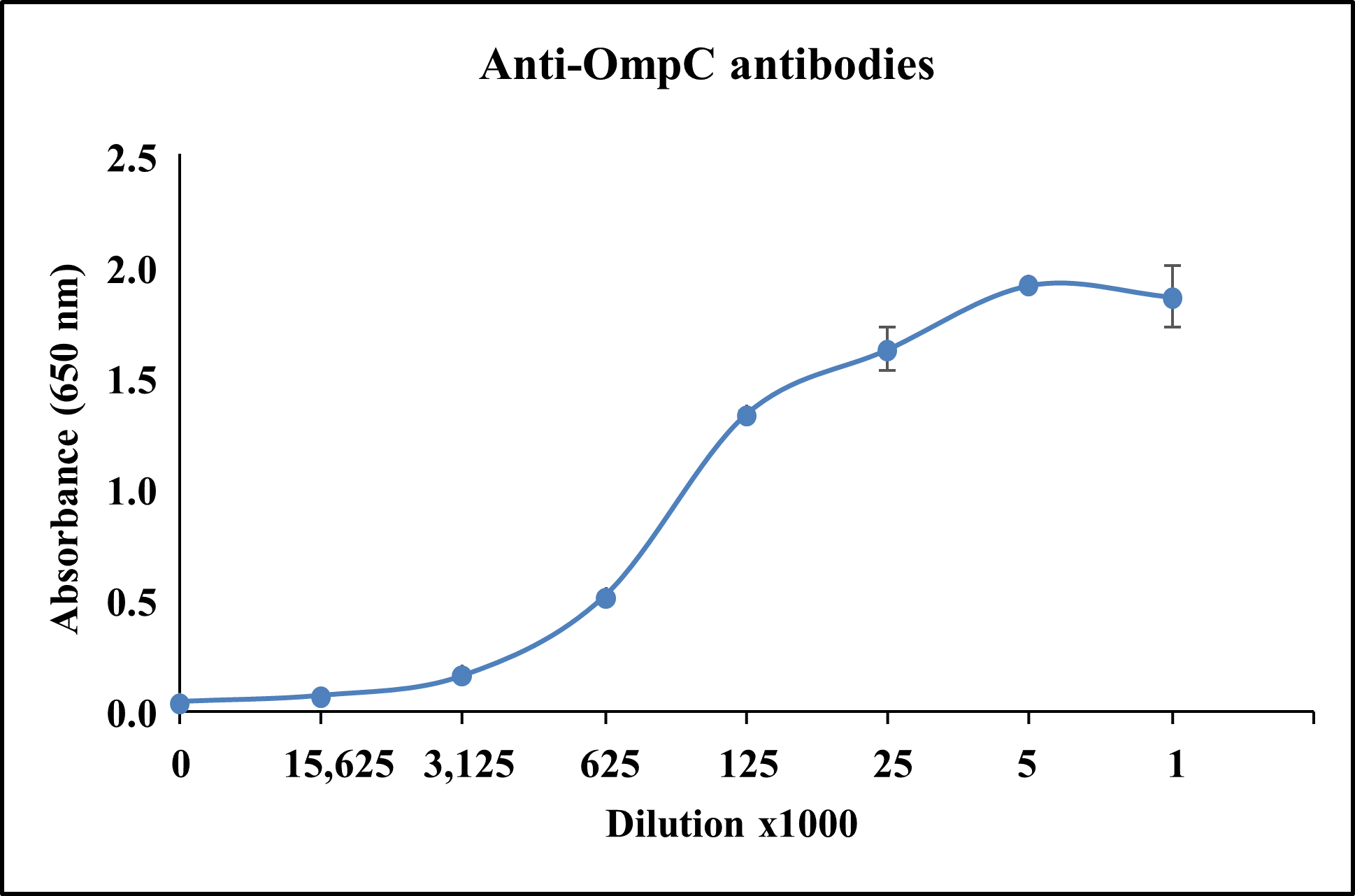

Supplement: S2 Fig — Anti-OmpC antibodies were produced by immunizing mice with the purified OmpC trimer. The endpoint titer was determined by applying serially diluted immune sera into the wells of a 96-well ELISA plate coated with purified OmpC. (TIF) [file ppat.1008193.s002.tif]

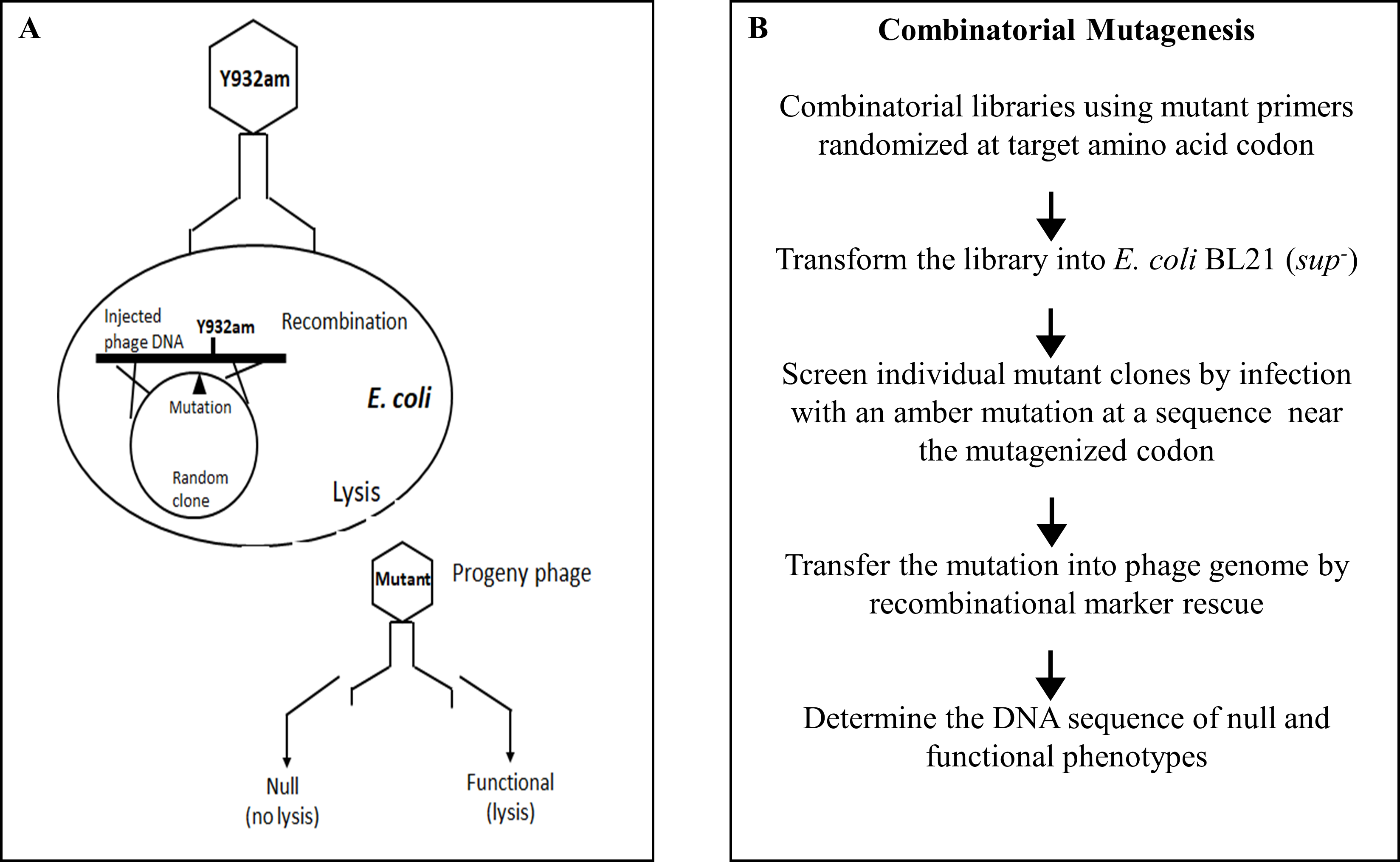

Supplement: S3 Fig — (A, B) Schematics showing the construction of libraries by introducing all possible codons at a given amino acid and transferring each mutation into phage genome by recombination using an amber mutation (e.g., Y932am) at a nearby flanking amino acid. The phenotype, functional or null, of the resultant progeny phage was determined by their ability to form plaques on an appropriate E. coli strain. The mutation giving rise to a phenotype is determined by PCR amplification of mutant DNA and DNA sequencing. (TIF) [file ppat.1008193.s003.tif]
